# Effect of Vitamin B_12_ and Folic Acid Supplementation on Bone Mineral Density and Quantitative Ultrasound Parameters in Older People with an Elevated Plasma Homocysteine Level: B-PROOF, a Randomized Controlled Trial

**DOI:** 10.1007/s00223-015-9968-6

**Published:** 2015-02-25

**Authors:** Anke W. Enneman, Karin M. A. Swart, Janneke P. van Wijngaarden, Suzanne C. van Dijk, Annelies C. Ham, Elske M. Brouwer-Brolsma, Nikita L. van der Zwaluw, Rosalie A. M. Dhonukshe-Rutten, Tischa J. M. van der Cammen, Lisette C. P. G. M. de Groot, Joyce van Meurs, Paul Lips, André G. Uitterlinden, M. Carola Zillikens, Natasja M. van Schoor, Nathalie van der Velde

**Affiliations:** 1 Department of Internal Medicine, Erasmus MC, University Medical Center Rotterdam, P.O. Box 2040, 3000 CA Rotterdam, The Netherlands; 2 Department of Epidemiology and Biostatistics and the EMGO Institute for Health and Care Research, VU University Medical Center, Van der Boechorststraat 7, 1081 BT Amsterdam, The Netherlands; 3 Division of Human Nutrition, Wageningen University, P.O. Box 8129, 6700 EV Wageningen, The Netherlands; 4 Department of Endocrinology, VU University Medical Center, P.O. Box 7057, 1007 MB Amsterdam, The Netherlands; 5 Department of Internal Medicine, Section of Geriatric Medicine, Academic Medical Center, P.O. Box 22700, 1100 DD Amsterdam, The Netherlands

**Keywords:** DXA, QUS, Vitamin B_12_, Folic acid, Homocysteine

## Abstract

High plasma homocysteine (Hcy) levels are associated with increased osteoporotic fracture incidence. However, the mechanism remains unclear. We investigated the effect of Hcy-lowering vitamin B_12_ and folic acid treatment on bone mineral density (BMD) and calcaneal quantitative ultrasound (QUS) parameters. This randomized, double-blind, placebo-controlled trial included participants aged ≥65 years with plasma Hcy levels between 12 and 50 µmol/L. The intervention comprised 2-year supplementation with either a combination of 500 µg B_12_, 400 µg folic acid, and 600 IU vitamin D or placebo with 600 IU vitamin D only. In total, 1111 participants underwent repeated dual-energy X-ray assessment and 1165 participants underwent QUS. Femoral neck (FN) BMD, lumbar spine (LS) BMD, calcaneal broadband ultrasound attenuation (BUA), and calcaneal speed of sound (SOS) were assessed. After 2 years, FN-BMD and BUA had significantly decreased, while LS-BMD significantly increased (all *p* < 0.01) and SOS did not change in either treatment arm. No statistically significant differences between the intervention and placebo group were present for FN-BMD (*p* = 0.24), LS-BMD (*p* = 0.16), SOS (*p* = 0.67), and BUA (*p* = 0.96). However, exploratory subgroup analyses revealed a small positive effect of the intervention on BUA at follow-up among compliant persons >80 years (estimated marginal mean 64.4 dB/MHz for the intervention group and 61.0 dB/MHz for the placebo group, *p* = 0.04 for difference). In conclusion, this study showed no overall effect of treatment with vitamin B_12_ and folic acid on BMD or QUS parameters in elderly, mildly hyperhomocysteinemic persons, but suggests a small beneficial effect on BUA in persons >80 years who were compliant in taking the supplement.

## Introduction

Approximately a decade ago, plasma levels of homocysteine (Hcy) were discovered to be positively associated with incident osteoporotic fractures [1, 2]. Vitamin B_12_ and/or folate are important co-factors in the remethylation of Hcy to methionine, and high plasma Hcy levels are often caused by vitamin B_12_ and/or folate deficiency [3]. Subsequent supplementation with these vitamins has been shown to be effective in reducing levels of Hcy [4]. Supplementation was, therefore, hypothesized to be associated with a lower fracture incidence as well. However, intervention studies with B-vitamin supplementation observed inconsistent effects on fracture prevention [5–8].

The potential mechanism underlying the association between Hcy and fractures remains to be determined. One of the hypotheses concerns the role of bone mineral density (BMD) in this association. Previously, cross-sectional studies on the relation between Hcy and BMD showed conflicting results (e.g., [9–11]). Moreover, two trials investigated the effect of B-vitamin supplementation on BMD, and both observed no effects [6, 12]. However, these trials were limited either in size (*n* = 47) [12] or in generalizability (hemiplegic post-stroke patients) [6] and both used fairly high doses of B-vitamins.

Alternatively, Hcy is thought to interfere with collagen cross-linking in bone, thereby reducing bone quality. This suggestion is supported by clinical observations in patients with homocystinuria, among whom bone collagen profiles are disturbed [13]. Previous cross-sectional data indeed observed inverse associations between Hcy and bone quality, as reflected by quantitative ultrasound (QUS) parameters [14–16]. However, intervention studies on the effect of B-vitamin supplementation on those QUS parameters are lacking.

The current study investigated the effects of vitamin B_12_ and folic acid supplementation on BMD and QUS parameters, that is broadband ultrasound attenuation (BUA) and speed of sound (SOS), in a large, mildly hyperhomocysteinemic, but otherwise general elderly population.

## Materials and Methods

### Study Design

The B-PROOF study is a double-blind, randomized, placebo-controlled multicenter trial. It was primarily designed to investigate the effect of 2-year oral supplementation with 400 µg folic acid and 500 µg vitamin B_12_ on osteoporotic fracture incidence in hyperhomocysteinemic persons aged 65 years and over [17]. Participants in both treatment arms additionally received 600 IU of vitamin D daily. Participants (*n* = 2919) were randomly assigned to the treatment groups in a 1:1 ratio while stratifying for study center, sex, age (65–80, ≥80 years), and Hcy level (12–18, ≥18 μmol/L). The random allocation sequence and randomization were generated and performed using SAS 9.2 by an independent research dietician. Intervention and placebo tablets were indistinguishable in taste, smell, and appearance. Both the participants and all researchers and research assistants were blinded to the study treatment. Treatment effects on BMD and QUS were predefined secondary outcomes of the B-PROOF study [17]. Recruitment of participants took place between September 2008 and March 2011. Details of the B-PROOF study were described previously [17]. The B-PROOF study has been registered with the Netherlands Trial Register http://www.trialregister.nl under identifier NTR 1333 since June 1, 2008 and with ClinicalTrials.gov under identifier NCT00696514 since June 9, 2008. The Medical Ethics Committee of Wageningen University (WU) approved the study and local feasibility was given by the Medical Ethics Committees of VU University Medical Center (VUmc) and Erasmus MC. The study was performed in accordance with the Declaration of Helsinki and all individual participants gave written informed consent.

### Study Population

**Fig. 1:**
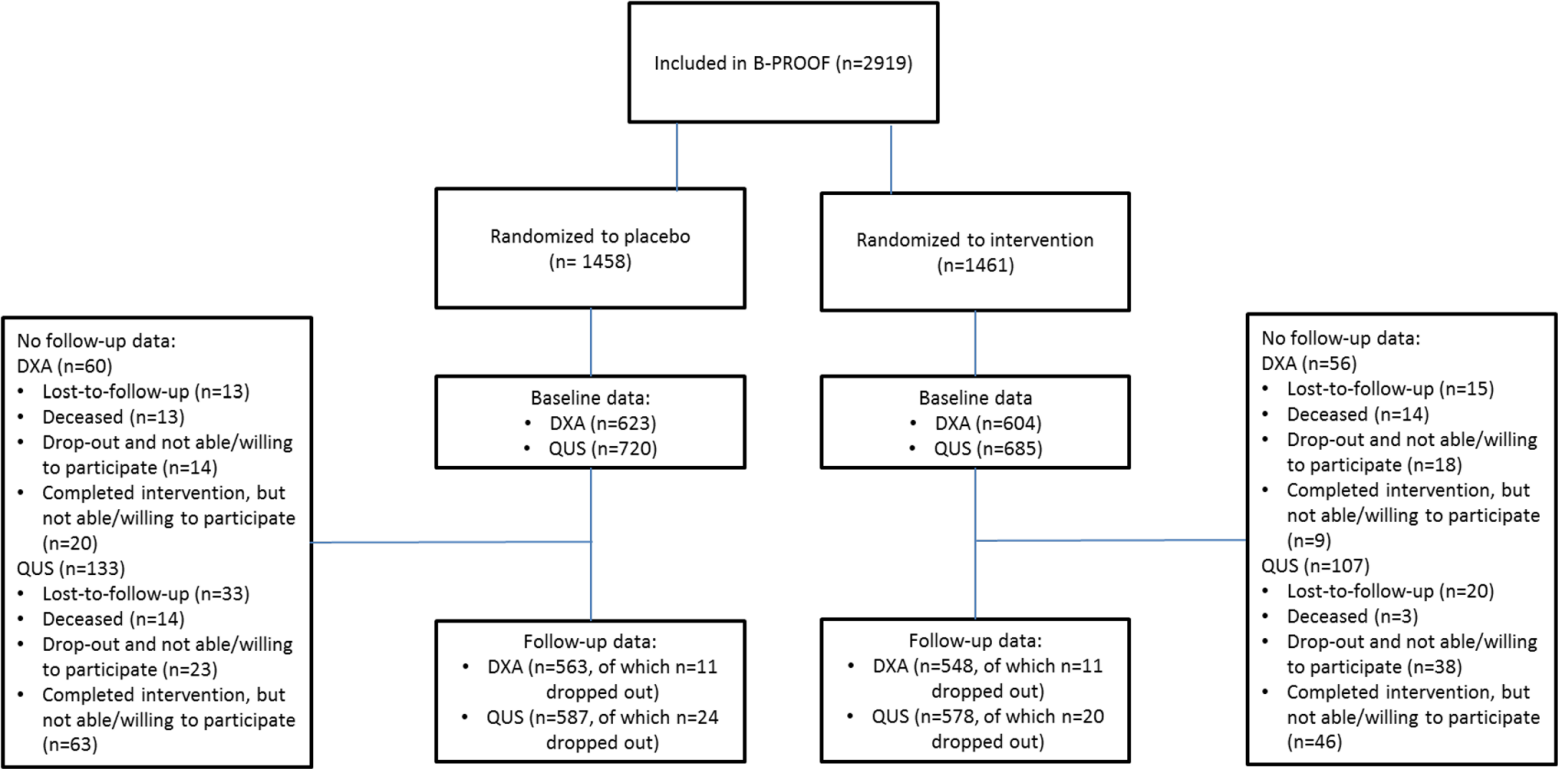
Flow-chart regarding DXA and QUS-measurements in the B-PROOF study

Inclusion criteria were an age of 65 years or over at baseline and a plasma Hcy level between 12.0 and 50.0 µmol/L. Exclusion criteria were a serum creatinine level >150 µmol/L, the presence of cancer in the past 5 years (excluding non-melanoma skin cancer), use of high doses of B-vitamins (intramuscular injections of vitamin B_12_ and/or folic acid intake >300 µg/day) or permanent use of a wheel chair. For BMD measurements, participants had to be able to visit one of the study centers. Figure 1 shows the flow-chart of the study sample.

### Basic Characteristics

At baseline, height was measured without shoes to the nearest millimeter using a stadiometer. Weight was measured while the participant wore light clothes and no shoes. Body mass index was calculated as weight/height^2^. Structured questionnaires were used to assess fracture history, current use of medication and supplements, level of education, use of alcohol, and current smoking behavior [17]. Anti-osteoporotic medication use was defined as the use of bisphosphonates, strontium ranelate, selective estrogen-receptor modulators, estrogens, androgens, denosumab, or teriparatide. Blood was withdrawn when the participant was in a fasted state or had consumed a light, restricted breakfast. EDTA-blood was placed on ice immediately after being withdrawn. Plasma Hcy, serum creatinine, folate, vitamin B_12_, holotranscobalamin, 25OH-vitamin D and methylmalonic acid, and methylenetetrahydrofolate reductase (MTHFR)-genotype were determined; details of the methods used have been described previously [8, 17].

### Dual-Energy X-ray (DXA) Assessment

In a subsample of 1227 participants, DXA was performed at baseline. Of these participants, 1111 persons also underwent a DXA after the 2 years of intervention (Fig. 1). DXA was performed in two of the three study centers. In VUmc, a Hologic QDR 4500 Delphi device (Hologic Inc., USA, CV = 0.45 %) was used. In Erasmus MC, a GE Lunar Prodigy device (GE Healthcare, USA, CV = 0.08 %) was used. A scan of the femur was made to determine the BMD at the femoral neck (FN). The left hip was scanned, but in case of a prosthesis, the right hip was scanned. A scan of the lumbar spine (LS) was made to assess BMD in the vertebrae L1 to L4. Measurements were performed according to the manufacturer’s protocols.

In Erasmus MC, during the intervention period, a new scanner of the same type was installed. Follow-up measurements for participants who were measured using the new device at follow-up were adjusted for results of a cross-calibration with the old system. A participant’s baseline and follow-up measurement always took place in the same study center.

### QUS Parameters

QUS parameters of the calcaneus were measured using the portable Hologic Sahara bone densitometer (Hologic, USA) (Erasmus MC, VUmc, WU) or the portable CUBA Clinical system (McCue Ultrasonics, UK) (VUmc). At baseline, QUS-measurements were performed in 1405 participants. Repeated QUS was available in 1165 participants (Fig. 1). Measurements of both the left and right calcaneus were performed in duplo. Mean broadband ultrasound attenuation (BUA, CV = 3.7 %) and speed of sound (SOS, CV = 0.22 %) were calculated as the average of these four measurements. Measurements were excluded if the expected linear frequency-attenuation relation was violated, because this indicates invalid results.

### Compliance

Participants were asked to return the remaining study tablets every 6 months during their 2-year intervention period. Participants were regarded as compliant to the study treatment when at least 80 % of the tablets had been taken during the intervention period, as indicated by the number of returned tablets. Compliance of participants who dropped out of the study was calculated over the planned full study period of 2 years.

### Adverse Events

Adverse events were reported by the participants on their study calendar or via telephone, as has been described previously [8].

### Sample Size Calculation and Statistical Analyses

Based on an expected increase in BMD of 0.027 g/cm^2^ (extrapolated from [18], who observed a 1-year-change in spinal BMD of 0.0135 when folate levels increased with 15 nmol/L) between the two treatment groups, an SD of 0.18 g/cm^2^ and a power of 80 % to detect this difference, we estimated that 541 participants had to be included in both treatment arms. Similarly, a decline in BUA of 2.1 dB/MHz is expected in 2 years in the placebo group, and we expect this decline to be prevented in the intervention group (extrapolated from [19]). With a difference of 2.1 dB/MHz and an SD of 9.4, 316 participants per group would be needed.

All statistical analyses were performed according to a predefined analysis plan. Differences in baseline characteristics between the two treatment groups were tested using a *t* test for continuous traits and a Chi-squared test for categorical traits. If a variable was non-normally distributed, a Mann–Whitney *U* test was used. Two-year changes in markers of B-vitamins (Hcy, folate, vitamin B_12_, methylmalonic acid, and holotranscobalamin) within treatment groups were tested using Wilcoxon signed-rank tests. Changes between treatment groups were tested with independent samples *t* tests.

In the primary intention-to-treat analyses, all participants of whom both baseline and follow-up data were available were included. In the secondary per-protocol analyses, only compliant participants were included. Paired *t* tests were done to assess the difference within treatment groups between baseline and follow-up for all outcomes. To test the difference in outcomes after 2 years of treatment between the intervention group and the placebo group, analysis of covariance (ANCOVA) was performed. In addition to the baseline value of the outcome of interest (FN-BMD, LS-BMD, BUA, or SOS), sex and age were entered as covariate in the basic model. This was defined as the primary analysis. Next, other potential confounders, defined by a *p*-value of the difference between the treatment arms <0.2, were entered in the model. They were retained in the fully adjusted model if they changed F of the treatment in the basic model with at least 10 %. This was done for each outcome separately. For BMD, analyses were repeated after stratification for study center, since both centers used different DXA-devices, which are known to produce systematically different results.

Interactions between treatment and baseline age, sex, and Hcy were investigated in exploratory analyses. Stratified analyses were performed if the interaction term was statistically significant. All statistical analyses were performed using IBM SPSS Statistics 20. Level of significance was set at *α* = 0.05.

## Results

Table 1 shows the general characteristics at baseline of 1111 participants with repeated DXA and of 1165 participants with repeated QUS. At baseline, LS-BMD was higher in the intervention group compared with the placebo group (1.14 vs 1.11 g/cm^2^, respectively, *p* = 0.03). In the BMD-sample, levels of serum holotranscobalamin were slightly higher in the intervention group (70 vs 65 µmol/L, *p* = 0.03). In the QUS-sample, participants in the placebo group more often had a positive fracture history (45 vs 35 %, *p* < 0.01).

**Table 1:** Baseline characteristics for B-PROOF participants with DXA at baseline and follow-up (*N* = 1111) and for participants with QUS at baseline and follow-up (*n* = 1165)

	BMD		QUS	
	Placebo	Intervention	Placebo	Intervention
	*N* = 563	*N* = 548	*N* = 587	*N* = 578
Age (years)^a^	72.8 (5.4)	72.4 (5.6)	73.3 (73.3)	73.4 (73.4)
Sex (% female)	48.3	48.2	57.4	53.8
Hcy (µmol/L)^b^	14.3 [12.9–16.3]	14.3 [12.9–16.0]	14.3 [12.9–16.4]	14.2 [13.0–16.1]
Creatinine (µmol/L)^b^	80 [71–93]	82 [71–93]	79 [70–92]	82 [70–93]
Folate (nmol/L)^b^	19.1 [14.8–25.4]	19.8 [15.4–24.8]	19.1 [14.8–24.5]	18.9 [15.6–24.6]
B_12_ (pmol/L)^b^	269 [204–343]	286 [218–348]	268 [204–352]	270 [216–346.3]
Methylmalonic acid (µmol/L)^b^	0.21 [0.17–0.29]	0.21 [0.17–0.28]	0.22 [0.18–0.30]	0.23 [0.18–0.30]
Holotranscobalamin (pmol/L)^b^	65 [47–88]*	70 [50–91]*	65 [45–85]	66 [49–88]
Vitamin D (25OH) (nmol/L)^b^	52.6 [37.1–70.3]	53.3 [37.2–73.0]	55.7 [39.2–72.4]	56.0 [37.4–75.1]
MTHFR-genotype (%)				
CC	43.1	47.9	43.2	47.4
CT	41.9	40.1	46.3	39.2
TT	15.0	12.0	10.5	13.4
Height (cm)^a^	169.9 (8.9)	170.4 (9.0)	168.5 (8.8)	168.9 (9.2)
Weight (kg)^a^	77.7 (12.9)	78.5 (13.0)	76.7 (12.2)	76.6 (12.5)
BMI (kg/m^2^)^a^	26.9 (3.9)	27 (3.8)	27.0 (3.9)	26.8 (3.8)
Smoking status (%)				
Current	8.7	8.4	7.5	10.0
Former	58.6	56.9	55.2	56.2
Never	32.7	34.7	37.3	33.7
Alcohol consumption (%)				
No/light	62.9	64.4	64.9	67.3
Moderate	31.8	31.2	30.7	28.4
Excessive	4.8	3.6	3.9	3.5
Very excessive	0.5	0.7	0.5	0.9
Level of education (%)				
Low	54.8	52.2	53.6	52.6
Middle	19.9	18.8	22.2	20.4
High	25.3	29.0	24.2	27.0
Study center (%)				
VUmc	35.7	32.5	35.4	36.2
Wageningen UR	–	–	20.4	21.1
Erasmus MC	64.3	67.5	44.1	42.7
Users of folic acid and/or vit. B_12_ (%)	17.1	14.6	17.4	14.4
Osteoporotic medication use (%)	6.4	7.5	8.9	10.4
Positive fracture history (%)	41.4	39.1	45.0*	35.3*
FN-BMD (g/cm^2^)^a^	0.84 (0.15)	0.85 (0.17)	–	–
*T*-score FN-BMD^a^	−1.23 (0.93)	−1.15 (1.04)	–	–
LS-BMD (g/cm^2^)^a^	1.11 (0.22)*	1.14 (0.25)*	–	–
*T*-score LS-BMD^a^	−0.3 (1.7)	−0.1 (1.9)	–	–
BUA (dB/MHz)^a^	–	–	70.9 (16.8)	71.8 (17.6)
SOS (m/s)^a^	–	–	1537 (31)	1539 (33)

*BMD* bone mineral density, *QUS* quantitative ultrasound, *BMI* body mass index, *FN* femoral neck, *LS* lumbar spine, *MTHFR* methylenetetrahydrofolate reductase

* *p*-value < 0.05

^a^Presented as mean (standard deviation)

^b^Presented as median [interquartile range]

A total of 611 participants had both FN-BMD as well as QUS available at baseline and at follow-up. At baseline, FN-BMD correlated significantly with both BUA (*r* = 0.48, *p* < 0.01) and SOS (*r* = 0.42, *p* < 0.01).

Changes in levels of Hcy, folate, vitamin B_12_, methylmalonic acid, and holotranscobalamin are shown in Table 2. Hcy changed significantly in the intervention group only. The other markers changed in both the intervention (improvements only) and placebo group (both improvements and deteriorations). *p* for differences in change between the groups was <0.001 for all markers, indicating that the compliance was good. Similar findings were observed in the QUS-sample.

**Table 2:** Baseline, follow-up, and change levels of B-vitamin markers in the B-PROOF DXA-sample

	Placebo					Intervention					*p* for difference in change
	*n* ^a^	Baseline^b^	Follow-up^b^	Change^c^	*p* for change	*n* ^a^	Baseline^b^	Follow-up^b^	Change^c^	*p* for change	
Hcy (µmol/L)	561	14.3 [12.9–16.3]	14.4 [12.7–16.9]	0.2 (3.8)	0.522	545	14.3 [12.9–16.0]	10.5 [9.2–12.0]	−4.2 (3.0)	<0.001	<0.001
Folate (nmol/L)	553	19.1 [14.8–25.3]	24.6 [20.0–31.4]	6.5 (9.9)	<0.001	541	19.8 [15.4–24.8]	51.7 [41.2–64.2]	33.3 (24.3)	<0.001	<0.001
Vitamin B_12_ (pmol/L)	553	268 [104–343]	289 [226–392]	70 (585)	<0.001	541	272 [218–348]	592 [461–736]	327 (186)	<0.001	<0.001
MMA (µmol/L)	551	0.21 [0.17–0.29]	0.23 [0.18–0.30]	0.02 (0.15)	<0.001	540	0.21 [0.17–0.28]	0.18 [0.15–0.22]	−0.07 (0.17)	<0.001	<0.001
HoloTC (pmol/L)	557	65 [47–88]	62 [44–82]	−4 (34)	<0.001	545	70 [50–91]	126 [95–180]	63 (54)	<0.001	<0.001

*MMA* methylmalonic acid, *HoloTC* holotranscobalamin

^a^Participants from the DXA-sample with both a baseline and follow-up determination of a marker were included

^b^Presented as median [interquartile range]

^c^Presented as mean (standard deviation)

### BMD Effects

**Fig. 2:**
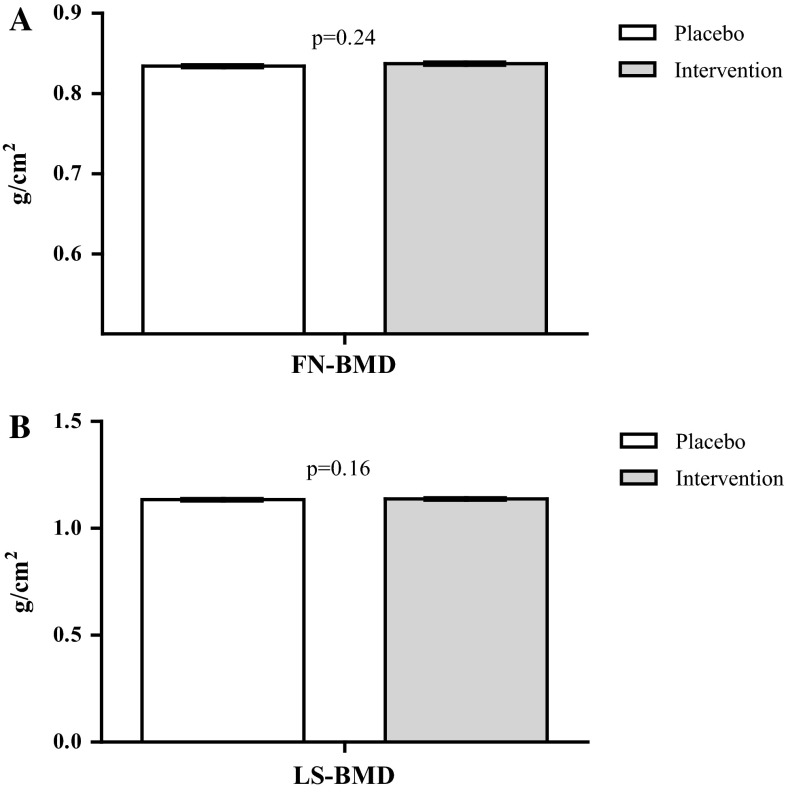
Estimated mean FN-BMD (**a**) and LS-BMD (**b**) after 2 years of intervention, adjusted for baseline FN-BMD/LS-BMD, age, and sex

Baseline and follow-up BMD per treatment group are shown in Table 3. FN-BMD significantly decreased in both treatment groups. On the contrary, LS-BMD increased significantly in both treatment groups. BMD in both the FN (0.84 g/cm^2^ (95 % CI 0.834–0.839) in the intervention group vs 0.83 g/cm^2^ (95 % CI 0.831–0.837) in placebo p = 0.24), and LS (1.14 g/cm^2^ (95 % CI 1.134–1.142) vs 1.13 g/cm^2^ (95 % CI 1.130–1.138), respectively, *p* = 0.16) were not significantly different between treatment groups (Fig. 2). This did not change after adjusting for other potential confounders (holotranscobalamin and vitamin B_12_). No statistically significant interaction was observed. When the analyses were stratified for study center, as pre-specified, similar results were obtained. For FN-BMD, in VUmc, estimated means after 2 years were 0.717 (95 % CI 0.712–0.722) and 0.719 (95 % CI 0.714–0.724) g/cm^2^ in the placebo and intervention groups, respectively. In Erasmus MC, these values were 0.896 (95 % CI 0.892–0.899) and 0.898 (95 % CI 0.895–0.902) g/cm^2^, respectively. For LS-BMD, in VUmc, estimated means after 2 years were 1.018 (95 % CI 1.011–1.024) and 1.017 (95 % CI 1.010–1.024) g/cm^2^ in the placebo and intervention groups, respectively. In Erasmus MC, corresponding values were 1.202 (95 % CI 1.197–1.207) and 1.208 (95 % CI 1.203–1.212) g/cm^2^. All differences were non-significant.

**Table 3:** Bone mineral density (*n* = 1111) and quantitative ultrasound parameters (*n* = 1165) at baseline and follow-up

	Placebo			Intervention		
	Baseline	Follow-up	*p*-value	Baseline	Follow-up	*p*-value
FN-BMD (g/cm^2^)	0.84 (0.15)	0.83 (0.15)	<0.01	0.85 (0.17)	0.85 (0.17)	<0.01
LS-BMD (g/cm^2^)	1.11 (0.22)	1.12 (0.22)	<0.01	1.14 (0.29)	1.15 (0.25)	<0.01
BUA (dB/MHz)	70.9 (16.8)	68.5 (17.4)	<0.01	71.8 (17.6)	69.4 (17.9)	<0.01
SOS (m/s)	1537 (31)	1537 (33)	0.25	1540 (34)	1539 (35)	0.46

*FN* femoral neck, *LS* lumbar spine, *BMD* bone mineral density, *BUA* broadband ultrasound attenuation, *SOS* speed of sound. Presented as mean (standard deviation)

In the per-protocol analyses, 1069 participants were included, and results were similar to the intention-to-treat analyses (data not shown).

### QUS Effects

**Fig. 3:**
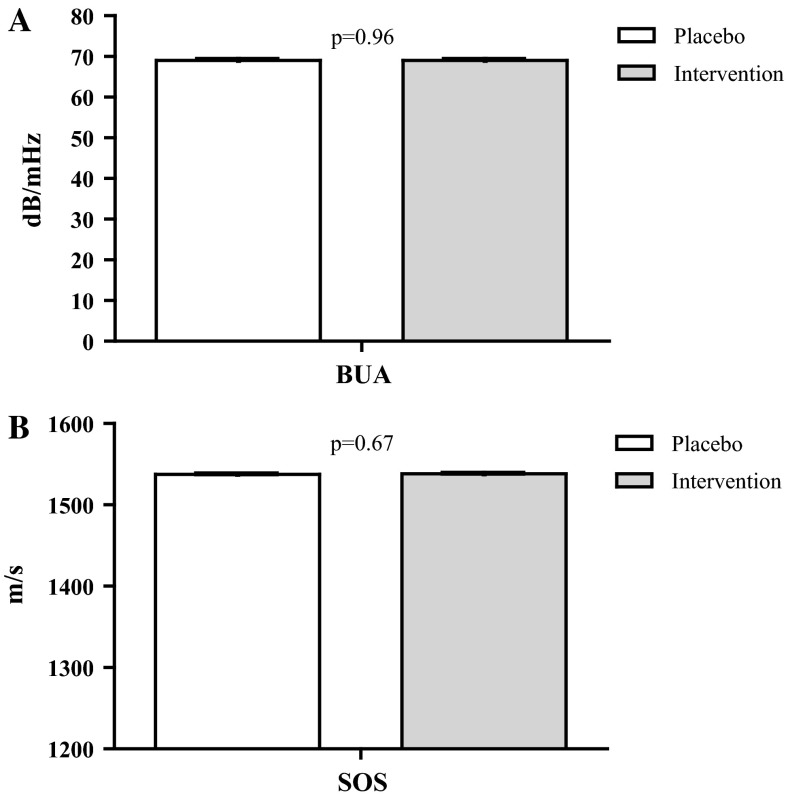
Estimated mean BUA (**a**) and SOS (**b**) after 2 years of intervention, adjusted for baseline BUA/SOS, age, and sex

A significant 2-year decline in BUA was observed in both the intervention group and the placebo group (both *p* < 0.01), whereas SOS levels did not change significantly in any of the groups (Table 3). Changes in BUA and SOS were not significantly different between treatment groups after adjustments for age, sex, and baseline values of BUA/SOS (Fig. 3a, b). The estimated marginal means for BUA were 69.0 dB/MHz (95 % CI 68.4–69.6) in both the intervention group and in the placebo group (*p* = 0.96), and the estimated marginal means for SOS were 1538.1 m/s (95 % CI 1536.6–1539.6) in the intervention group versus 1537.6 m/s (95 % CI 1536.2–1539.1) in the placebo group (*p* = 0.67). Additional adjustments for fracture history, holotranscobalamin, smoking, vitamin B supplement use and MTHFR-genotype (BUA), or fracture history, smoking, and MTHFR-genotype (SOS) did not change the findings (data not shown). No interactions with age, sex, and baseline Hcy concentration were observed.

**Fig. 4:**
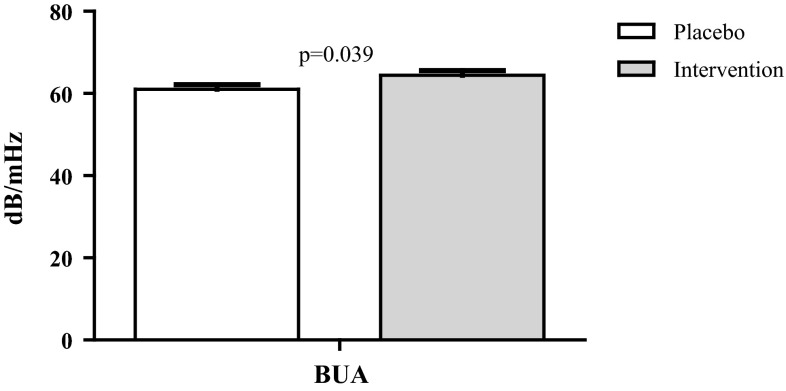
Estimated mean BUA among compliant persons >80 years after 2 years of intervention, adjusted for baseline BUA, age, and sex

Results of the per-protocol analyses, including 1097 participants, did not substantially differ from the intention-to-treat analyses (data not shown). Yet, in the analyses with BUA as outcome, the interaction with age was significant (*p* = 0.02). Exploratory, stratified analyses showed no effect among persons ≤80 years, but among persons >80 years, a significant beneficial effect of the treatment was observed (*p* = 0.04, Fig. 4). The estimated marginal means were 64.4 dB/MHz (95 % CI 62.1–66.6) in the intervention group versus 61.0 dB/MHz (95 % CI 58.8–63.3) in the placebo group.

## Discussion

This randomized controlled trial did not show an overall effect of 2-year oral folic acid and vitamin B_12_ supplementation on BMD and QUS parameters compared with the placebo. In a subgroup of persons >80 years who were compliant with the study protocol, a small but statistically significant positive effect of the B-vitamin intervention was observed on BUA.

This study is the first trial investigating the effects of vitamin B_12_ and folic acid on QUS. Moreover, effects on BMD have not been studied before in a large, mildly hyperhomocysteinemic, but otherwise general older population. Two previous trials have been conducted, showing results that are in concordance with our findings. A Japanese trial investigated the effect of 1.5 mg vitamin B_12_ and 5 mg folic acid on hip fracture incidence and metacarpal BMD in hemiplegic post-stroke patients. In that study, no effect of a 2-year treatment on BMD was observed, while fracture incidence was strongly and significantly reduced in this specific population [6]. In addition, a small trial (*n* = 47) has been performed which investigated the effect of a 1-year treatment with vitamin B_12_, B_6_, and folic acid on BMD among osteoporotic patients [12]. Overall, no effects were observed in that study. However, in participants with Hcy >15 µmol/L (*n* = 8 in the intervention group), a significant increase in *T*-score was seen. In our study, no interaction effect of the treatment with baseline Hcy levels was observed. It should be noted that in comparison to our study, Herrmann et al. used higher doses (2.5 mg folic acid, 25 mg B_6_, and 500 µg B_12_) [12].

QUS parameters are largely determined by BMD, but bone microarchitecture is an important determinant as well, independent of BMD [20]. QUS has been shown to be an independent predictor for fracture risk [21]; a decrease of 1 SD in BUA has been associated with a 1.4 fold increased risk of any clinical fracture [21]. We observed a mean difference in BUA of 3.4 dB/MHz (5.2 % of mean baseline BUA) between the intervention and placebo group among compliant persons >80 years. The observed difference between the groups was larger than the coefficient of variation (5.2 vs 3.4 %). However, because the spreading of BUA is relatively large (SD = 17.1), the observed effect will be of minor importance on population level. However, when applying a longer duration of intervention, it might become clinically relevant.

We observed an effect on BUA in the subgroup of compliant persons >80 years, but no effect on SOS was observed. Although the correlation between BUA and SOS is strong (*R* = 0.7), their measurement is based on different constructs. In addition, they have been shown to be influenced by a different set of independent determinants [22]. These differences may explain the different effects on BUA and SOS. However, a chance finding cannot be ruled out either.

Recently, we have shown within the B-PROOF study that fracture incidence was lower in the intervention group compared with placebo only when specifically addressing compliant participants aged 80 years or over [8]. The currently reported change in BUA might partly explain this age-specific treatment effect, and supports the suggestion of the role of homocysteine in bone collagen cross-linking. Unfortunately, we were not able to test the hypothesis of BUA as mediator in the age-specific treatment effect on fractures, due to a too low absolute number of fractures among participants >80 years of whom BUA data were available (*n* = 23). Alternatively, the lack of an effect on BMD does not completely rule out the possibility of BMD as a mediator. Participants of the DXA-subsample had to be able to visit one of the study centers and may therefore not be fully representative of the complete study population: as compared to the total sample, the DXA-subsample was significantly younger (mean age 72.6 vs 74.1, *p* < 0.01), with a lower percentage of persons aged >80 years (9.0 vs 16.9 %, *p* < 0.01). In line with this, the subgroup of persons aged >80 years with DXA was also significantly younger than the subgroup of the complete study population (mean age 83.9 vs 85.1, *p* < 0.01). The somewhat selective sample hampers definite conclusions about the absence of an effect of B-vitamins on BMD in persons >80 years.

It should be noted that LS-BMD increased in both treatment groups during 2 years of intervention, while FN-BMD decreased. In older persons, an increase in LS-BMD can be expected due to, for instance, degenerative changes of the spine [23, 24]. Our observation therefore supports the presumption that LS-BMD may not be a valid indicator of osteoporosis at high age [25]. It could be regarded as a limitation that baseline levels of BMD in this randomized controlled trial differed significantly between the intervention and placebo group. However, we adjusted for baseline BMD, and therefore we assume that this did not influence the results of the analyses. Another limitation of the study is the fact that all participants received 600 IU vitamin D daily, which is in line with the guidelines of the Dutch Health Council [26]. In the past, vitamin D supplementation with 400 IU daily has been shown to influence BMD up to 2.6 % [27, 28]. Effects of vitamin D may therefore have masked the possibly small effects of vitamin B_12_ and folic acid on BMD.

From the current study, we conclude that there is no overall effect of 2-year treatment with vitamin B_12_ and folic acid on BMD or QUS in hyperhomocysteinemic elderly people. Among elderly >80 years who were compliant in taking the supplement, a positive effect of the treatment on BUA was observed. This might partly explain the previously reported reduction in fracture risk in the same subgroup [8]. It is important to note that an adverse effect of our treatment with vitamin B_12_ and folic acid on cancer incidence was observed, as has been published previously [8], implying caution in designing further research. Nonetheless, research on effects of B-vitamin treatment on other mechanisms, for instance on bone markers, computed tomography, or potentially the relatively new assessment of trabecular bone score, is warranted to reveal the additional pathways by which vitamin B_12_ and folic acid exert a potential anti-fracture effect in hyperhomocysteinemic elderly.

## References

[1] McLean RR, Jacques PF, Selhub J (2004). Homocysteine as a predictive factor for hip fracture in older persons. N Engl J Med.

[2] van Meurs JB, Dhonukshe-Rutten RA, Pluijm SM (2004). Homocysteine levels and the risk of osteoporotic fracture. N Engl J Med.

[3] Selhub J, Jacques PF, Wilson PW, Rush D, Rosenberg IH (1993). Vitamin status and intake as primary determinants of homocysteinemia in an elderly population. JAMA.

[4] Homocysteine Lowering Trialists C (2005). Dose-dependent effects of folic acid on blood concentrations of homocysteine: a meta-analysis of the randomized trials. Am J Clin Nutr.

[5] Gommans J, Yi Q, Eikelboom JW, Hankey GJ, Chen C, Rodgers H (2013). The effect of homocysteine-lowering with B-vitamins on osteoporotic fractures in patients with cerebrovascular disease: substudy of VITATOPS, a randomised placebo-controlled trial. BMC Geriatr.

[6] Sato Y, Honda Y, Iwamoto J, Kanoko T, Satoh K (2005). Effect of folate and mecobalamin on hip fractures in patients with stroke: a randomized controlled trial. JAMA.

[7] Sawka AM, Ray JG, Yi Q, Josse RG, Lonn E (2007). Randomized clinical trial of homocysteine level lowering therapy and fractures. Arch Intern Med.

[8] Van Wijngaarden JP, Swart KMA, Enneman AW (2014). Effect of daily vitamin B12 and folic acid supplementation on fracture incidence in elderly individuals with an elevated plasma homocysteine level: B-PROOF, a randomized controlled trial. Am J Clin Nutr.

[9] Baines M, Kredan MB, Usher J (2007). The association of homocysteine and its determinants MTHFR genotype, folate, vitamin B12 and vitamin B6 with bone mineral density in postmenopausal British women. Bone.

[10] Gjesdal CG, Vollset SE, Ueland PM (2006). Plasma total homocysteine level and bone mineral density: the Hordaland Homocysteine Study. Arch Intern Med.

[11] Perier MA, Gineyts E, Munoz F, Sornay-Rendu E, Delmas PD (2007). Homocysteine and fracture risk in postmenopausal women: the OFELY study. Osteoporos Int.

[12] Herrmann M, Umanskaya N, Traber L (2007). The effect of B-vitamins on biochemical bone turnover markers and bone mineral density in osteoporotic patients: a 1-year double blind placebo controlled trial. Clin Chem Lab Med.

[13] Lubec B, Fang-Kircher S, Lubec T, Blom HJ, Boers GH (1996). Evidence for McKusick’s hypothesis of deficient collagen cross-linking in patients with homocystinuria. Biochim Biophys Acta.

[14] Dhonukshe-Rutten RA, Pluijm SM, de Groot LC, Lips P, Smit JH, van Staveren WA (2005). Homocysteine and vitamin B12 status relate to bone turnover markers, broadband ultrasound attenuation, and fractures in healthy elderly people. J Bone Miner Res.

[15] Gerdhem P, Ivaska KK, Isaksson A (2007). Associations between homocysteine, bone turnover, BMD, mortality, and fracture risk in elderly women. J Bone Miner Res.

[16] Enneman AW, Swart KM, Zillikens MC (2014). The association between plasma homocysteine levels and bone quality and bone mineral density parameters in older persons. Bone.

[17] van Wijngaarden JP, Dhonukshe-Rutten RA, van Schoor NM (2011). Rationale and design of the B-PROOF study, a randomized controlled trial on the effect of supplemental intake of vitamin B12 and folic acid on fracture incidence. BMC Geriatr.

[18] Cagnacci A, Bagni B, Zini A, Cannoletta M, Generali M, Volpe A (2008). Relation of folates, vitamin B12 and homocysteine to vertebral bone mineral density change in postmenopausal women. A five-year longitudinal evaluation. Bone.

[19] Krieg MA, Jacquet AF, Bremgartner M, Cuttelod S, Thiebaud D, Burckhardt P (1999). Effect of supplementation with vitamin D3 and calcium on quantitative ultrasound of bone in elderly institutionalized women: a longitudinal study. Osteoporos Int.

[20] Cortet B, Boutry N, Dubois P, Legroux-Gerot I, Cotten A, Marchandise X (2004). Does quantitative ultrasound of bone reflect more bone mineral density than bone microarchitecture?. Calcif Tissue Int.

[21] Moayyeri A, Adams JE, Adler RA, Krieg M-A, Hans D, Compston J, Lewiecki EM (2012). Quantitative ultrasound of the heel and fracture risk assessment: an updated meta-analysis. Osteoporos Int.

[22] Canhao H, Lucas R, Fonseca JE, Costa L, Romeu JC, Branco J, Barros H (2008). Factors influencing calcaneus quantitative ultrasound measurements in an urban population. Clin Exp Rheunatol.

[23] Masud T, Langley S, Wiltshire P, Doyle DV, Spector TD (1993). Effect of spinal osteophytosis on bone mineral density measurements in vertebral osteoporosis. BMJ.

[24] Wang Y, Boyd SK, Battie MC, Yasui Y, Videman T (2011). Is greater lumbar vertebral BMD associated with more disk degeneration? A study using microCT and discography. J Bone Miner Res.

[25] Richtlijn Osteoporose en Fractuurpreventie (2011). derde herziening.

[26] Gezondheidsraad. Evaluatie van de voedingsnormen voor vitamine D (2012) The Hague: Gezondheidsraad

[27] Dawson-Hughes B, Dallal GE, Krall EA, Harris S, Sokoll LJ, Falconer G (1991). Effect of vitamin D supplementation on wintertime and overall bone loss in healthy postmenopausal women. Ann Intern Med.

[28] Ooms ME, Roos JC, Bezemer PD, van der Vijgh WJ, Bouter LM, Lips P (1995). Prevention of bone loss by vitamin D supplementation in elderly women: a randomized double-blind trial. J Clin Endocrinol Metab.

